# Anteromedial Portal Reference Technique for Femoral Tunnel Depth Measurement During Arthroscopic Anterior Cruciate Ligament Reconstruction

**DOI:** 10.7759/cureus.13147

**Published:** 2021-02-05

**Authors:** Gunjar Jain, Rameshwar Datt, Asjad Mahmood, Hira Lal Nag, Arpit Sahu

**Affiliations:** 1 Orthopaedics, All India Institute of Medical Sciences, New Delhi, IND

**Keywords:** knee, arthroscopy, anterior cruciate ligament reconstruction

## Abstract

Femoral tunnel preparation during the anterior cruciate ligament reconstruction is a technically demanding step. The anteromedial (AM) portal technique necessitates knee hyperflexion during the femoral tunnel reaming. In a hyperflexed knee, the arthroscopic visualization of the laser markings on the femoral tunnel reamer becomes obscured. Thus, the calculation of the depth of the femoral tunnel becomes difficult. Our technique helps in femoral tunneling reliably without the need for arthroscopic visualization using the AM portal as a reference point to calculate the depth while drilling. This technique can be performed without the need for a second assistant to hold the arthroscope. Furthermore, this technique does not require any specific instruments, and there is no obligation for an additional incision.

## Introduction

The anteromedial (AM) portal technique is a popular method to create the femoral tunnel during arthroscopic anterior cruciate ligament (ACL) reconstruction. With this method, one can determine the precise anatomical femoral tunnel position. It is critical to prepare the femoral tunnel of an exact pre-estimated depth. A deeper tunnel may cause a blowout of the femoral tunnel, while a shorter one can complicate the deployment of the endobutton or the interference screw.

The AM portal technique has limitations as the arthroscopic visibility often gets compromised during the technique [1]. Multiple factors result in poor visibility. First, there is an inadequate flow of the arthroscopic fluid because of knee flexion. Second, the bone debris coming out of the tunnel while drilling also hinders the view. Finally, the infra-patellar fat pad ingresses into the joint while advancing the reamer and blocks the vision [2]. Thus, it becomes difficult to visualize the laser markings over the femoral reamer. Furthermore, we need two assistants for this technique, one to hold the arthroscope and the other for maintaining knee hyperflexion. With the AM portal reference technique, we can prepare the femoral tunnel via the AM portal avoiding all these obstacles.

## Technical report

The surgical technique is described for ACL reconstruction using hamstring tendon autograft fixed with an endobutton on the femoral side and an interference screw on the tibial side. The patient is positioned supine on the operative table with a lateral post and a foot bump such that the knee can be moved freely through the full range of motion (ROM) and can be rested at 90° flexion. A thigh tourniquet is applied as proximal as possible. After preparation and draping of the limb, the tourniquet is inflated, and a standard anterolateral portal is made. A diagnostic arthroscopy is performed, and any concurrent intraarticular pathology is addressed. The working AM portal is made medial to the patellar tendon just above the medial meniscus under arthroscopic vision. The ideal site for femoral tunnel preparation is marked with a bone awl at the center of the femoral ACL footprint. A guidewire is passed via the AM portal through femoral ACL footprint and exited from the lateral femoral condyle with the knee in the hyperflexed position. A starter reamer of size 4.5 mm is passed over the guidewire again in knee hyperflexion, and the total length (TL) of the femoral tunnel is measured.

The semitendinosus graft is harvested. The graft is prepared using an appropriate size endobutton device on the femoral side. We commonly use an endobutton with a loop size (LS) of either 15 or 20 mm depending upon the TL of the femoral tunnel, such that a graft tunnel overlap of at least 20 mm is obtained. The thickness of the graft is measured, and it is then tensioned to reduce creeping after fixation. Then, the required depth of femoral tunnel (FT) over-drilling is estimated using the formula FT = TL - LS + 10.

**Figure 1:**
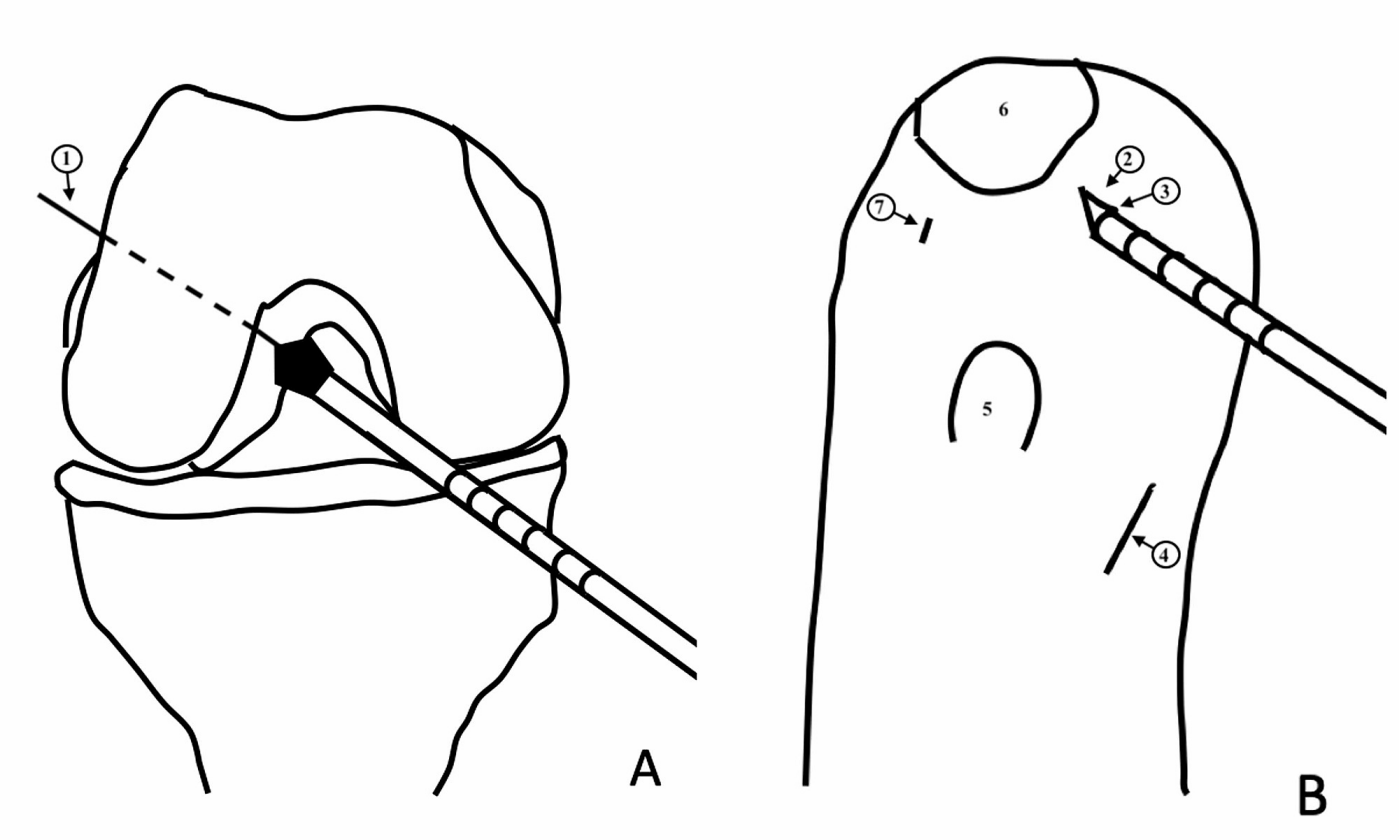
Schematic diagram to demonstrate the AM portal reference technique. The femoral tunnel reamer is passed through the AM portal over the guidewire and is rested over the medial aspect of the lateral femoral condyle (A). The calibration marking of the reamer coinciding with the AM portal is noted (B). AM: anteromedial 1: guidewire; 2: AM portal; 3: calibration mark over the reamer coinciding with the AM portal; 4: incision site for hamstring tendon graft harvest; 5: tibial tuberosity; 6: patella; 7: anterolateral portal

**Figure 2:**
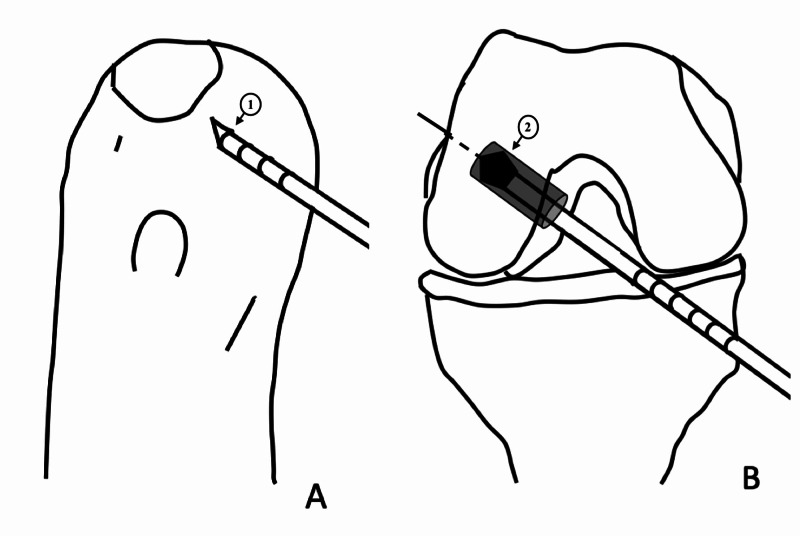
Schematic diagram to demonstrate the AM portal reference technique on the right knee. The femoral tunnel reamer is advanced over the guidewire until the pre-decided laser mark coincides with the AM portal (A). This creates the femoral tunnel of the desired depth without requiring a direct visualization (B). AM: anteromedial 1: pre-decided mark over the reamer coinciding with the AM portal; 2: femoral tunnel of the desired depth

**Figure 3:**
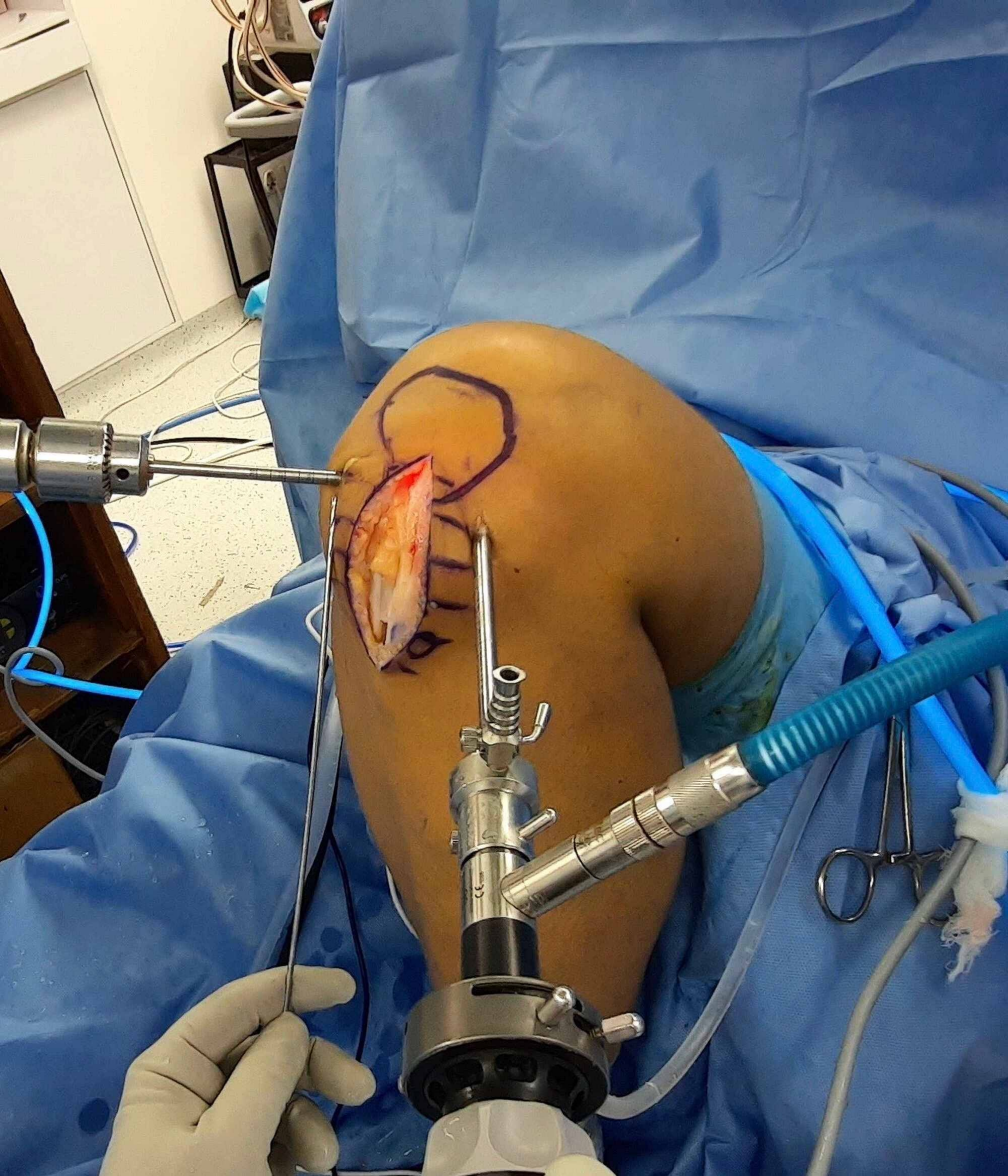
A 30-year-old male patient undergoing arthroscopic ACL reconstruction of the left knee with bone-patellar tendon-bone graft. The image shows the preparation of the femoral tunnel via the AM portal reference technique. Before reaming, the arthroscope in the anterolateral portal is used to visualize the adjacent structures and confirm their safety. The guidewire is pointing towards the AM portal. The AM portal is used as a reference to measure the depth of the femoral tunnel. ACL: anterior cruciate ligament; AM: anteromedial

Finally, the guidewire is passed through the femoral tunnel. An appropriate size drill bit depending upon the graft thickness is advanced over the guidewire and rested over the bone. Subsequently, the knee is moved to full flexion. At this point, one can remove the arthroscope from the joint if no additional assistant is available. Thus, the femoral tunnel can be created with only one assistant. Because this technique involves drilling without visualization, before withdrawing the arthroscope from the joint, it must be ensured that no other nearby structures are damaged during the procedure. The laser mark of the reamer lying against the AM portal site is noted (Figure 1). The determined length of the femoral tunnel over-drilling is then added to this number. The reamer is advanced until the specific laser marking reaches up to the portal site (Figure 2). Finally, the reamer is removed from the joint and the knee is extended to 90° flexion. The arthroscope is introduced into the joint to visualize the femoral tunnel. The rest of the steps, including tibial tunnel preparation and graft passage and fixation, are performed conventionally. This technique can be adopted for different grafts such as the bone-patellar tendon-bone graft (Figure 3).

## Discussion

Femoral tunnel preparation during the ACL reconstruction is a technically demanding step. The AM portal technique necessitates knee hyperflexion during the femoral tunnel reaming. However, hyperflexion of the knee obscures the arthroscopic picture. In such cases, if we extend the knee to improve the visualization, it can direct the tunnel posteriorly and cause a posterior tunnel blow-out [2]. Nevertheless, special techniques to prepare the femoral tunnel keeping the knee in a lesser degree of flexion have been described by many authors. For instance, Steiner et al. proposed to use a flexible guide-pin and reamer to perform femoral socket preparation in less knee flexion via the AM portal [3]. The outside-in technique also avoids hyperflexion of the knee; however, this technique requires additional skin incision over the lateral aspect of the distal thigh [1]. Lately, a retrograde drilling device has been introduced to avoid this extra skin incision [4]. However, the retrograde drilling device or the flexible guide-pin and reamer are not available in a traditional setting and increase the cost of the procedure.

Our technique of femoral tunnel preparation does not require any specific device. Thus, the technique is cost-effective. One more advantage is that a second assistant is not required in this technique. As the assistant surgeon maintains the knee flexion, the chief surgeon removes the arthroscope and performs the drilling. Thus, we can create the femoral tunnel through the AM portal using our technical modification in two situations. First, when no additional assistance is available, and, second, in cases where it is difficult to visualize the reamer in a hyperflexed knee. The principal disadvantage to our technique is the risk of injury to adjacent structures, which can be avoided by examining the reaming site before withdrawing the arthroscope. Therefore, this modified technique should be used only when arthroscopic visibility of the knee is compromised (Table 1).

Furthermore, as it is an indirect method, and the reference point can be altered with changing knee positions, the accuracy of measurement can be compromised. However, we commonly use this method of femoral tunnel reaming. The depth of the tunnel created is accurate every time, and we have never faced any difficulty during the deployment of the fixation device (Table 2). We have also not encountered any other complication such as injuries to any nearby structures or femoral tunnel blow-out.

**Table 1:** Advantages and disadvantages of the proposed technique.

Advantages	Disadvantages
No need of visualizing the laser mark of the femoral reamer arthroscopically, which can be difficult and time consuming	Technically demanding to ensure the protection of adjacent structures while reaming
Does not require additional assistance while preparing the femoral tunnel	As it is an indirect method of measurement, its accuracy might be compromised
Does not require special devices and reduces the cost of surgery	Change in knee position may alter the positioning of the portal site with reference to the reamer

**Table 2:** Tips and pearls and pitfalls of the proposed technique.

Tips and pearls	Pitfalls
Starting point of reaming should be determined only after hyperflexing the knee	Before the withdrawal of the arthroscope and reaming of the femoral tunnel, a proper evaluation of proximity of the nearby structures is essential
Avoid any alteration of the relationship of the portal site and the reamer while reaming	The reamer should be withdrawn from the joint before extending the knee

## Conclusions

The AM portal reference technique helps in preparation of the femoral tunnel reliably without the need for arthroscopic visualization using the AM portal as a reference point to calculate the depth while drilling. It should be used cautiously with proper precautions to avoid any complications in patients where the arthroscopic visibility is compromised after hyperflexing the knee joint.

## References

[1] Burnham JM, Malempati CS, Carpiaux A, Ireland ML, Johnson DL (2017). Anatomic femoral and tibial tunnel placement during anterior cruciate ligament reconstruction: anteromedial portal all-inside and outside-in techniques. Arthrosc Tech.

[2] Lubowitz JH (2009). Anteromedial portal technique for the anterior cruciate ligament femoral socket: pitfalls and solutions. Arthroscopy.

[3] Steiner ME, Smart LR (2012). Flexible instruments outperform rigid instruments to place anatomic anterior cruciate ligament femoral tunnels without hyperflexion. Arthroscopy.

[4] Branam BR, Hasselfeld KA (2013). “Retrograde technique” for drilling the femoral tunnel in an anterior cruciate ligament reconstruction. Arthrosc Tech.

